# Imaging modalities for diagnosis and monitoring of cancer cachexia

**DOI:** 10.1186/s13550-021-00834-2

**Published:** 2021-09-23

**Authors:** Jessie Han, Luke Harrison, Lisa Patzelt, Mingming Wu, Daniela Junker, Stephan Herzig, Mauricio Berriel Diaz, Dimitrios C. Karampinos

**Affiliations:** 1 Department of Diagnostic and Interventional Radiology, Klinikum Rechts Der Isar, TUM School of Medicine, Technical University of Munich, Ismaninger Str. 22, 81675 Munich, Germany; 2 Institute for Diabetes and Cancer, Helmholtz Center Munich, 85764 Neuherberg, Germany; 3 German Center for Diabetes Research (DZD), 85764 Neuherberg, Germany; 4 Joint Heidelberg-IDC Translational Diabetes Program, Inner Medicine 1, Heidelberg University Hospital, Heidelberg, Germany; 5 Chair of Molecular Metabolic Control, Technical University of Munich, Munich, Germany

**Keywords:** Imaging biomarkers, Computed tomography (CT), Magnetic resonance imaging (MRI), Cancer cachexia progression, Imaging-based phenotyping, Skeletal muscle, Adipose tissue

## Abstract

Cachexia, a multifactorial wasting syndrome, is highly prevalent among advanced-stage cancer patients. Unlike weight loss in healthy humans, the progressive loss of body weight in cancer cachexia primarily implicates lean body mass, caused by an aberrant metabolism and systemic inflammation. This may lead to disease aggravation, poorer quality of life, and increased mortality. Timely detection is, therefore, crucial, as is the careful monitoring of cancer progression, in an effort to improve management, facilitate individual treatment and minimize disease complications. A detailed analysis of body composition and tissue changes using imaging modalities—that is, computed tomography, magnetic resonance imaging, (^18^F) fluoro-2-deoxy-d-glucose (^18^FDG) PET and dual-energy X-ray absorptiometry—shows great premise for charting the course of cachexia. Quantitative and qualitative changes to adipose tissue, organs, and muscle compartments, particularly of the trunk and extremities, could present important biomarkers for phenotyping cachexia and determining its onset in patients. In this review, we present and compare the imaging techniques that have been used in the setting of cancer cachexia. Their individual limitations, drawbacks in the face of clinical routine care, and relevance in oncology are also discussed.

## Introduction

Cachexia is a multifactorial wasting disorder associated with neoplastic diseases such as cancer and other, mainly chronic, diseases. Cancer cachexia is characterized by a substantial loss of body weight, including muscle wasting and, but not necessarily, adipose tissue loss. These features are driven by disturbances in protein, carbohydrate, and lipid metabolism and are associated with a systemic inflammatory state, conferring a negative energy balance [1, 2]. Simultaneously, cachexia-associated anorexia further exacerbates an already catabolic state, accelerating disease progression. Unlike starvation, a key feature of cancer cachexia is the inability to fully treat involuntary weight loss with standard nutritional support therapies [3], highlighting a critical energy homeostatic and metabolic disruption.

Cancer cachexia is classified into three linear stages: (1) Precachexia describes the early stage of the disease where minor weight loss changes occur that may present with prior glucose intolerance and anorexia. (2) Cachexia describes a body weight loss of > 5% within 6 months, a weight loss of > 2% in patients with a BMI of < 20, or sarcopenia. (3) Refractory cachexia describes the state at which reversibility of the disease, given the current day treatment strategies, is dramatically decreased, and average life expectancies reach below 3 months, with tumor treatments remaining unresponsive [3]. Progression through these stages may, however, vary from patient to patient, imparting the complexity of the underlying mechanisms and potential phenotypic regulators [2].

The heterogeneous presentation of cachexia in cancer is a culmination of multiple interplaying factors. This has hindered a clear consensus regarding clinical description in the past, leading to the frequent omission of cachexia-related deaths in national statistical databases, occluding prevalence and clinical relevance data [2]. The vast majority of data originates from nutritional screens conducted in national cancer centers [4–7]. However, the differing scores and selection criteria hamper clear readouts when combining outcomes. Cancer cachexia rates, given the nature of the disease, are comparable to those of untreatable terminal illnesses and are thus uniformly high toward end of life. Importantly though, cachexia must not always develop in late-stage cancer; this is supported by reports of skeletal muscle anabolism in patients with advanced-stage cancer [8, 9]. Certain cancers are also known to be more likely associated with cachexia: the cachexia incidence for prostate and breast cancer lies at approximately 20%, around 40–50% for hematological, colorectal and lung cancer, while gastroesophageal and pancreatic cancers exhibit alarmingly high rates ranging from 60 to 70% [4, 5]. The amount of weight patients will typically lose parallels the occurrence rates concerning cancer type, with approximately 2% body weight loss in prostate cancer, but up to 15% in pancreatic cancer [4, 5].

The high prevalence rate of cancer cachexia calls for cancer patient treatment frameworks which incorporate adequate cachexia management as well as transparent documentation of therapy outcomes. The dramatic sarcopenia, anorexia and fatigue experienced by patients have a severely detrimental impact on their quality of life [10]. This extends to family members and loved ones, who visually experience the ineffectiveness of treatment strategies and the decline in well-being of the patient, provoking frustration and fear, ultimately further decreasing the patient’s quality of life [11]. Involuntary weight loss is commonly the initial clinical presentation of cachexia, and thus it is critical that weight is carefully monitored after cancer diagnosis, as precachexia may reveal itself before weight loss begins [12]. The decrease in skeletal muscle mass often has devastating outcomes regarding cancer prognosis and outcome. An elevated chemotherapy toxicity has been noted in cachectic patients, resulting in reduced dosing, therapy delay or complete abandonment [10]. Furthermore, low body weight is known to dampen success rates after cancer surgery, increasing complications and risk of mortality [13, 14].

Despite an ever-increasing research base and support from the pharmaceutical industry, cancer cachexia largely remains an untreatable and unnoticed entity. With 20–30% of cancer deaths resulting from cachexia alone [15], the necessity to develop treatments, but also suitable methods for early detection are vital when looking ahead.

## Cancer cachexia as a multi-organ syndrome

**Fig. 1:**
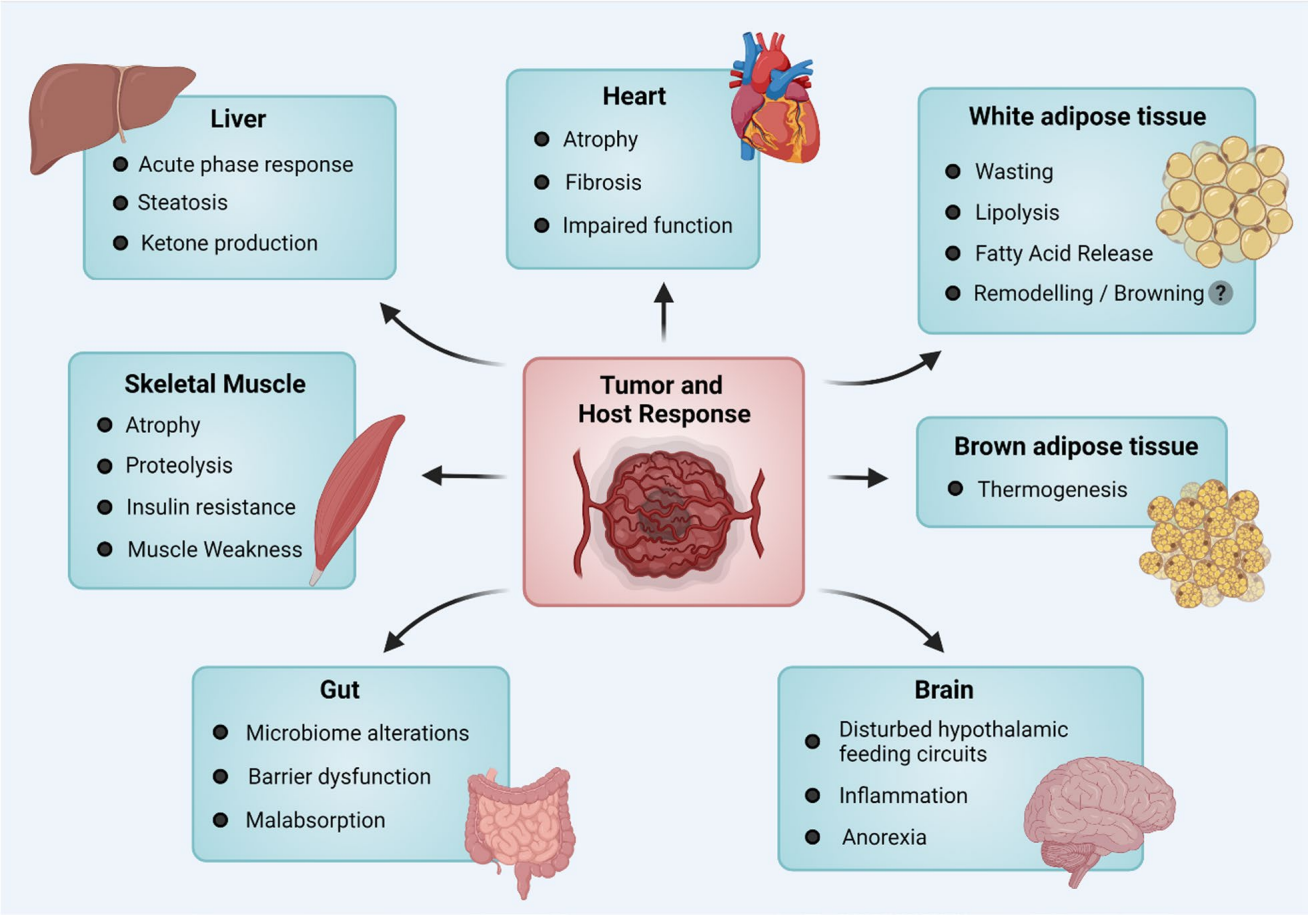
Schematic highlighting cancer cachexia as a multi-organ syndrome: Cancer cachexia is regulated by signals that are released from the primary tumor, but also by mechanisms initiated by the host response. These pathways involve a wide range of organs, of which the main ones are indicated here. While the classical cachexia organs such as skeletal muscle and the fat depots have been in focus for quite some time, other major tissues such as the liver, the heart, the gut, and the brain are now known to be impacted or involved (as listed by the bullet points) in this syndrome, and are making their way into the spotlight

In recent years, research focused on understanding the etiology of cancer cachexia has begun to shift from a muscle-focused field to a multi-organ view, as increasing evidence reveals a complex network of organ participation [16, 17]. Figure 1 provides a schematic overview of the tissues and organs affected by cancer cachexia, beyond skeletal muscle changes, including the liver, adipose tissue depots, the brain, the myocardium and the gut.

First, systemic inflammation drives the communication in this network through inflammatory factors derived from both the tumor as well as other organs in the network itself. The liver plays a key role in the systemic inflammatory state of cachexia via the acute phase response (APR) [18, 19]. Beyond inflammation, the induced hepatic APR may also contribute to energy dissipation with additional futile cycles such as the conversion of tumor-derived lactate into glucose via hepatic gluconeogenesis. These processes can be fueled by amino acids derived from muscle protein degradation [20]. Furthermore, hepatic triglyceride handling is impaired in cachectic models [21], which may further aggravate insulin resistance and muscle wasting [22].

Second, the composition and distribution of adipose tissue is altered in cancer cachexia. On the one hand, induced lipolysis contributes to the loss of white adipose tissue (WAT), which can precede muscle wasting [23]. Fat mass was a significant predictor of survival in these patients [23], emphasizing the importance of lipid stores in WAT as markers for health and energy status during cancer cachexia. Brown adipose tissue (BAT), known for its thermogenic potential, has more recently gained attention, as different studies have found evidence for increased uncoupling protein 1 (UCP1; a main driver of thermogenesis in adipose tissue and energy dissipation) expression in the WAT of cachectic mice and patients [24, 25]. However, this observation is not consistent in all models of cancer cachexia [26], and to which extent UCP-1-dependent thermogenesis or other cycles such as lipolysis and re-esterification cycling of fatty acids [16, 20] contribute to energy wasting in cancer cachexia, remains to be determined.

Third, the brain conduces pathogenically increased energy expenditure. While inflammation of the hypothalamus (a common topic in obesity research) may blunt food intake via a decreased appetite [27], the brain also has the capacity to stimulate lipolysis, WAT browning and to drive thermogenesis via sympathetic innervation of the adipose tissue [28].

Fourth, there is a convincing amount of evidence linking gut homeostasis to cancer cachexia. These links range from gut barrier dysfunction—due to epithelial layer breakdown and consequent systemic inflammation through invading pathogens—to an altered gut microbiome [16, 17].

Finally, cancer cachexia affects cardiac muscle tissue, leading to cardiac tissue atrophy and dysfunction [29–31]. Indeed, cardiac dysfunction is one of the underlying causes of death in cancer patients [32].

To summarize, cancer cachexia is a complex systemic disease comprised of an intricate network of signals from multiple organs. Currently, body weight remains the standard diagnostic tool in clinics for cachexia detection, yet if detection timescales are to improve, newer, more sensitive methods are required.

## Imaging in cancer cachexia

Since cancer cachexia constitutes a multi-organ syndrome that alters body composition and tissue quality over time, and given that many noninvasive imaging modalities can simultaneously assess these longitudinal changes, medical imaging holds the greatest potential in improving the phenotyping of cachexia developing cancer patients. Expanding the role of noninvasive imaging-based phenotyping could improve the efficacy of diagnosing cancer cachexia, anticipating high-risk individuals, systematically assessing the multi-organ effects of therapeutic interventions and enhancing our understanding of cancer cachexia pathophysiology. We provide below a short overview of the main technical characteristics of imaging modalities available for assessing tissue changes in cancer cachexia:

### Dual-energy X-ray absorptiometry (DXA)

Dual-energy X-ray absorptiometry (DXA) is a widely available, fast and inexpensive two-dimensional (2D) projection technique suitable to assess body composition by estimating body fat, lean tissue mass and bone mineral density [44]. In the context of cancer cachexia, DXA has been used to measure whole-body and regional distribution of skeletal muscle and WAT.

### Computed tomography (CT)

Computed tomography (CT) is a cross-sectional radiological imaging technique that is routinely practiced as part of the clinical staging of cancer patients. It is therefore the most commonly used imaging modality to assess body composition changes in cancer patients [64]. The discrimination of lean tissue from adipose tissue and their mixtures is performed with CT using the differences in attenuation coefficients of X-rays of these tissues. CT attenuation or radiodensity, expressed in Hounsfield units (HU), helps to determine the tissue lipid concentration and has been related to risk of disease progression and recurrence [103]. Specifically in the context of cancer cachexia, CT is suitable for quantifying whole body, regional and individual volume change of skeletal muscle and WAT. It can also determine the regional and individual distribution of fat depots. CT can further be applied to assess myosteatosis, the fatty infiltration of skeletal muscle, by measuring the mean muscle tissue radiodensity, known to be linearly dependent on skeletal muscle fat fraction.

### Magnetic resonance imaging (MRI)

**Fig. 2:**
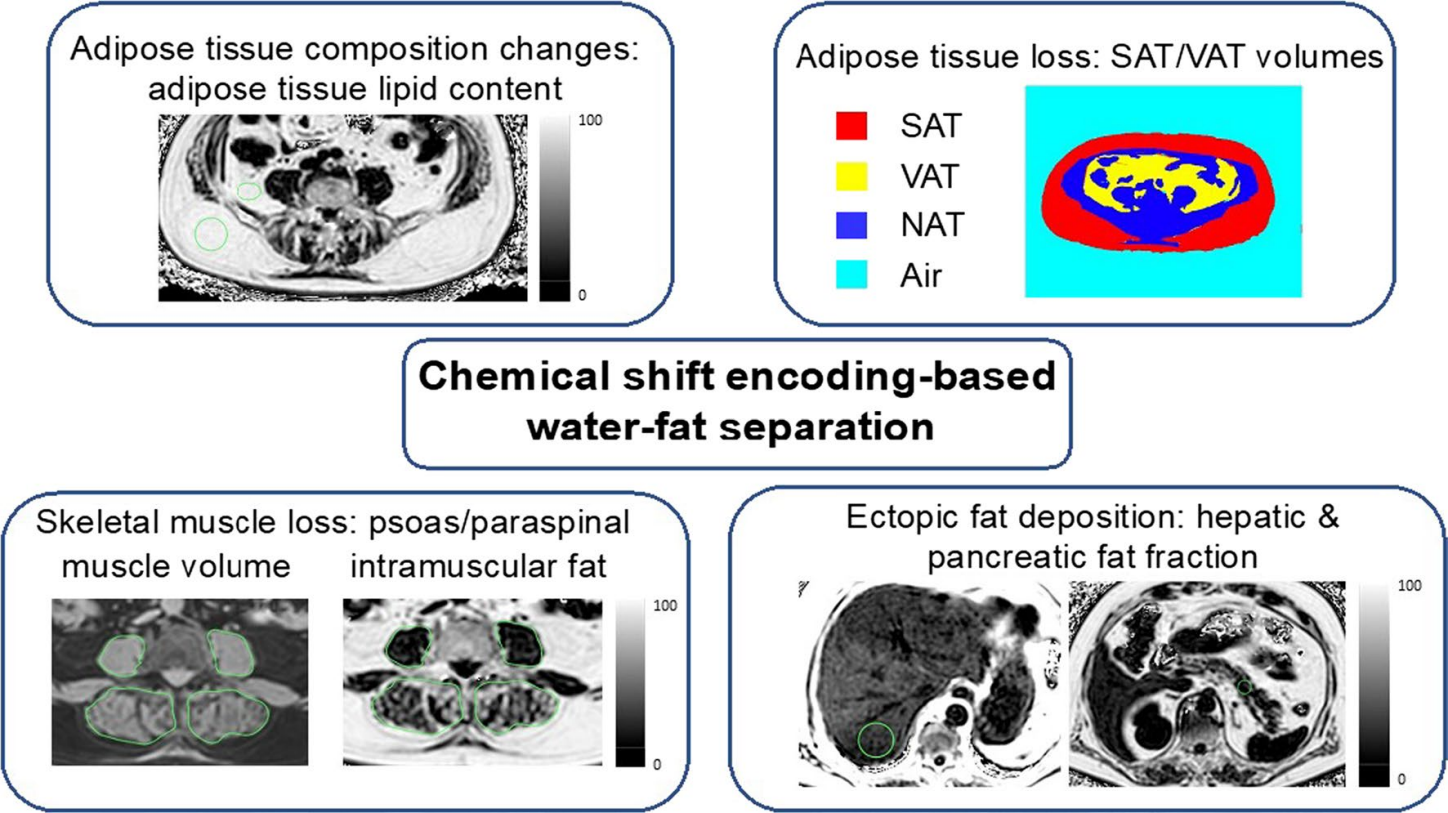
Schematic showing the possible MRI biomarkers extracted from an abdominal chemical shift encoding-based water-fat separation MR imaging acquisition, including the determination SAT/VAT volume, ectopic lipid content in the liver and pancreas, paraspinal muscle volume and intramuscular fat content and adipose tissue lipid content

Magnetic resonance imaging (MRI) is a cross-sectional radiological imaging technique, which, although widely adopted in the diagnostic setting, is not part of routine clinical staging in oncology. However, MRI is gaining significant attention for noninvasively assessing patients with metabolic dysfunction. MRI provides volumetric images without the burden of ionizing radiation and is therefore very attractive for longitudinally assessing tissues changes in patients with metabolic diseases undergoing lifestyle interventions [33, 34]. The richness of MR contrast mechanisms allows for qualitative and quantitative assessment of adipose tissue and muscle. Modern body composition MRI techniques rely on the use of chemical shift encoding-based water-fat separation methods. Water-fat MRI can quantify tissue lipid concentration in a standardized manner by calculating the proton density fat fraction (PDFF). It enables the simultaneous assessment of skeletal muscle volume and fat infiltration, of WAT volume, lipid content and of ectopic (liver, pancreas) lipid concentration (Fig. 2). In cancer cachexia cohorts, MRI has been primarily employed to assess changes in skeletal muscle volume and fat infiltration.

### Positron emission tomography (PET)

Positron emission tomography (PET) is a Nuclear Medicine imaging modality which applies radioactive tracers to measure tissue changes in metabolism and other physiological activities. (^18^F) fluoro-2-deoxy-d-glucose (^18^FDG) PET is being clinically routinely used to detect lesions and to measure the metabolic activity of tumors by quantifying the standardized uptake value (SUV). In cancer cachexia, ^18^FDG-PET enables imaging the metabolic activity of both the primary tumor and other affected tissues. By mapping the metabolic activity of primary tumors, researchers could relate this to cachexia progression. In addition, ^18^FDG-PET has been used to map white and BAT metabolic activity in cancer patients.

## Literature search

An electronic search in PubMed (http://www.ncbi.nlm.nih.gov/pubmed) was performed without a starting date up to May 2021 using as search terms “cancer cachexia” and one of the following terms: “Computed Tomography” “Magnetic Resonance Imaging”, “Positron Emission Tomography”, “Dual-energy X-ray absorptiometry”, “Imaging”. The search resulted in 488 entries, which included investigations in both animals and humans. The reference lists of relevant articles were also screened. The present review focuses on imaging modalities for diagnosis and monitoring of cancer cachexia used primarily in human studies. We have structured the review below based on the use of the imaging modalities for assessing changes in different tissue types, including imaging the primary tumor, imaging the skeletal muscle, imaging the WAT, and imaging the BAT.

## Imaging the primary tumor

The metabolic activity of the primary tumor and its metastases has been investigated with ^18^FDG-PET. Although the primary tumors are often small in size, their energy metabolism is upregulated, as tumor cells have been shown to switch to a less efficient metabolism in order to proliferate, exhibiting high rates of glycolysis [35, 36]. It has been postulated however that the glucose uptake measured in ^18^FDG-PET imaging is mainly driven by uptake into so-called cancer-associated fibroblasts, not into the tumor cells themselves [37, 38].

In a preclinical study in mice, ^18^FDG-PET analysis revealed an increased ^18^FDG uptake in cachexia-inducing tumors compared to non-cachectic tumors [39], confirming the primary tumor’s role in the development of the deranged energy metabolism in cancer. In a small human study in patients with esophageal cancer, Mitamura et al. found significant positive correlations between glucose uptake on ^18^FDG-PET and energy expenditure measured via indirect calorimetry [40], again pointing to the involvement of tumor metabolism in the energy imbalance in cancer patients. Furthermore, a study in gastric cancer patients found a positive correlation between metabolic tumor volume (i.e. the volume of the tumor with glucose uptake above a defined threshold) and the degree of weight loss the patients experienced [41].

Recent studies have gone beyond the simple analysis of mean ^18^FDG-PET uptake in tumors for predicting cachexia risk. A study in patients with advanced non-small-cell lung carcinoma found that a PET/CT-based radiomics analysis with focus on the primary tumor and skeletal muscle had the potential to predict the probability of developing cachexia before therapy begin [42]. In addition, in a previous clinical study in patients with lung cancer, Dolan et al. found elevated tumor metabolic activity, as measured by ^18^FDG-PET/CT imaging, to be associated with a greater risk of malnutrition, aside from a more advanced tumor stage, systemic inflammatory response and poorer survival. However, no correlation to body composition measured by CT was found [43].

In order to translate preclinical findings and findings from smaller clinical studies into the human metabolic environment, Friesen et al. modeled the energy demand of a tumor in vivo and found that tumor mass and the percentage of anaerobic metabolism in the tumor contribute to the energy burden caused by the tumor, which may lead to negative energy balance and increased muscle wasting [44]. However, as the processes involved in the development of cachexia are complex and the aforementioned clinical studies consist of mostly small sample sizes, further larger studies are needed to confirm the observations and help better understand to which extent the tumor’s energy consumption could contribute to metabolic imbalance and subsequent development of cachexia.

## Imaging the skeletal muscle

MRI and CT are currently considered the gold standard imaging modalities to assess muscle mass [45]. However, earlier cancer cachexia studies have also employed DXA techniques for assessing body composition in cancer patients. Since skeletal muscle has been the tissue studied most extensively using these imaging modalities, we review below how DXA, CT and MRI each have been utilized to assess skeletal muscle alterations in cancer cachexia.

### DXA of skeletal muscle in cancer cachexia

Total body lean mass as an estimation of whole-body muscle mass and appendicular lean mass as an estimation of skeletal muscle mass within the limbs are suitable measurements of body composition in cancer patients [46]. Therefore, DXA remains among the most commonly used methods to assess body composition in studies observing cachectic cancer patients [47]. DXA-derived measurements of appendicular lean tissue mass have been shown to highly correlate to both MRI and CT measures of skeletal muscle mass [48, 49]. However, it has been reported that DXA overestimates whole-body lean mass compared to MRI muscle mass measurements, which could lead to an underestimation of muscle loss [49, 50]. Such errors could be related to the employed assumptions on the DXA attenuation coefficients for the different tissue components (lean, fat and bone tissues).

A loss of muscle mass and body fat is a frequent observation when using DXA techniques in patients with advanced cancer [51, 52]. One study reported that 67% of cancer patients in palliative care had a low appendicular lean soft tissue index when assessed with DXA [53]. An accelerated depletion in body fat compared to lean tissue was reported [54]. Moreover, an uneven distribution of lean tissue mass reduction was found in the body: while lean tissue in the arm was lost, a gain in relative weight of lean tissue in trunk and legs was observed (*p* < 0.01). In this study, DXA-based lean tissue loss did not appear to be a significant predictor of survival.

Similar studies using DXA showed that appendicular lean mass can predict functional muscle performance in cancer patients [53, 55]. As other studies were unable to associate increased lean body mass (LBM) directly with increased physical function in cancer patients suffering from cachexia, the exact correlation when using DXA techniques is yet to be understood [56].

A sexual dimorphism regarding muscle mass and muscle depletion in cachectic cancer patients is known [57–59]. In a longitudinal study, it was found that loss of muscle mass progressed more rapidly in men than in women [51]. Also, the prevalence of skeletal muscle mass depletion was reported to be higher in men than in women.

### CT of skeletal muscle in cancer cachexia

Body composition analysis in cancer patients using CT is considered superior over DXA [60], as it provides tomographic data compared to only a 2D projection through the body. Skeletal muscle volume is generally evaluated with a cross-sectional CT analysis and single-slice measurements of paraspinal muscles have been shown to correlate with whole-body muscle mass [60, 61].

Sarcopenia, the loss of skeletal muscle mass, is frequent among cancer patients, is commonly associated with cachexia and leads to a decline in physical and strength performance [62]. Sarcopenia is associated with higher rates of mortality and morbidity in cancer patients [63–66]. Low skeletal muscle indices derived with CT correlated with higher levels of biomarkers of systemic inflammation such as CRP, Neutrophile Lymphocyte Ratio or albumin [67].

While functional performance and muscle mass were diminished in cancer patients suffering from cachexia, radiodensity was not reduced [55]. It has also been shown that protein content of skeletal muscle in cancer patients cannot be precisely estimated using muscle radiodensity measurements via CT [68]. In fact, it was observed that muscle radiodensity and muscle mass do not correlate significantly [64]. Others found that muscle radiodensity was a prognostic factor for survival. For example, a high radiodensity was associated with longer periods of survival in patients with non-small cell lung cancer [69]. Furthermore, muscle radiation attenuation, which is inversely related to muscle fat content, was an independent prognostic parameter for survival in cachexia of patients with epithelial ovarian cancer and low muscle attenuation was associated with a worsened nutritional and inflammatory status [70]. The predictive role of baseline skeletal muscle indices and muscle radiation attenuation in patients with gynecological malignancies remains an area of intense research, although the respective changes during the disease trajectory were found to correlate with survival [71].

The drastically diminished time period of survival among cachectic cancer patients suffering from muscle depletion and low muscle attenuation was found to be independent of individual BMI [72]. Low muscle attenuation, acquired as mean cross-sectional attenuation of the paraspinal muscles, was found to predict unsatisfactory therapy response in patients with metastatic renal cell carcinoma [73]. For various cancer types, low skeletal muscle indices were strongly associated with the prevalence of dose-limiting chemotherapy-related toxicity in patients undergoing chemotherapy [74–78]. It was observed that during chemotherapy, patients lost more muscle tissue whereas the intramuscular fat content increased [79]. These changes in body composition were adequately monitored by CT imaging, however were not representable when compared to changes in BMI or weight. Similarly, CT-derived measurements of muscle and adipose tissue demonstrated a stronger correlation to survival than BMI in breast cancer patients [80]. The loss of skeletal muscle in patients with advanced esophageal cancer undergoing neoadjuvant chemotherapy was predictive of postoperative mortality [81]. An increased risk of perioperative complications and worse long-term prognosis for sarcopenic lung cancer patients undergoing surgery was also observed [82].

There is strong evidence that CT and MRI measurements are equally suitable to assess biomarkers of skeletal muscle quality and quantity [83]. Myosteatosis, the fat infiltration of skeletal muscle, can be measured using either mean radiodensity (Hounsfield units) in CT or proton density fat fraction (PDFF, %) in MRI. A recent systematic review and meta-analysis described a shorter survival in cancer patients presenting with myosteatosis measured via CT [84]. Myosteatosis was directly associated with systemic inflammation in colorectal cancer patients [85]. Patients with higher levels of myosteatosis also had longer hospitalization times [86]. Finally, myosteatosis was associated with reduced survival in patients with pancreatic cancer and distal cholangiocarcinoma [87]. These studies indicate a negative association of myosteatosis with disease progression and survival. Notably though, the presence of myosteatosis without systemic inflammation has been correlated with longer progression-free survival and overall survival in patients with advanced esophageal cancer [88].

### MRI of skeletal muscle in cancer cachexia

Using MRI, the muscle tissue can be further investigated and contractile tissue volume and muscle fat volume can be obtained. MRI also enables the discrimination of intra- and intermuscular fat depots. However, as MRI is not typically part of the clinical staging routine of most cancer patients, it is not routinely used to assess body composition and cachexia in cancer patients. Therefore, little is in general known about MRI-derived measurements of cachexia-induced changes in body composition.

**Fig. 3:**
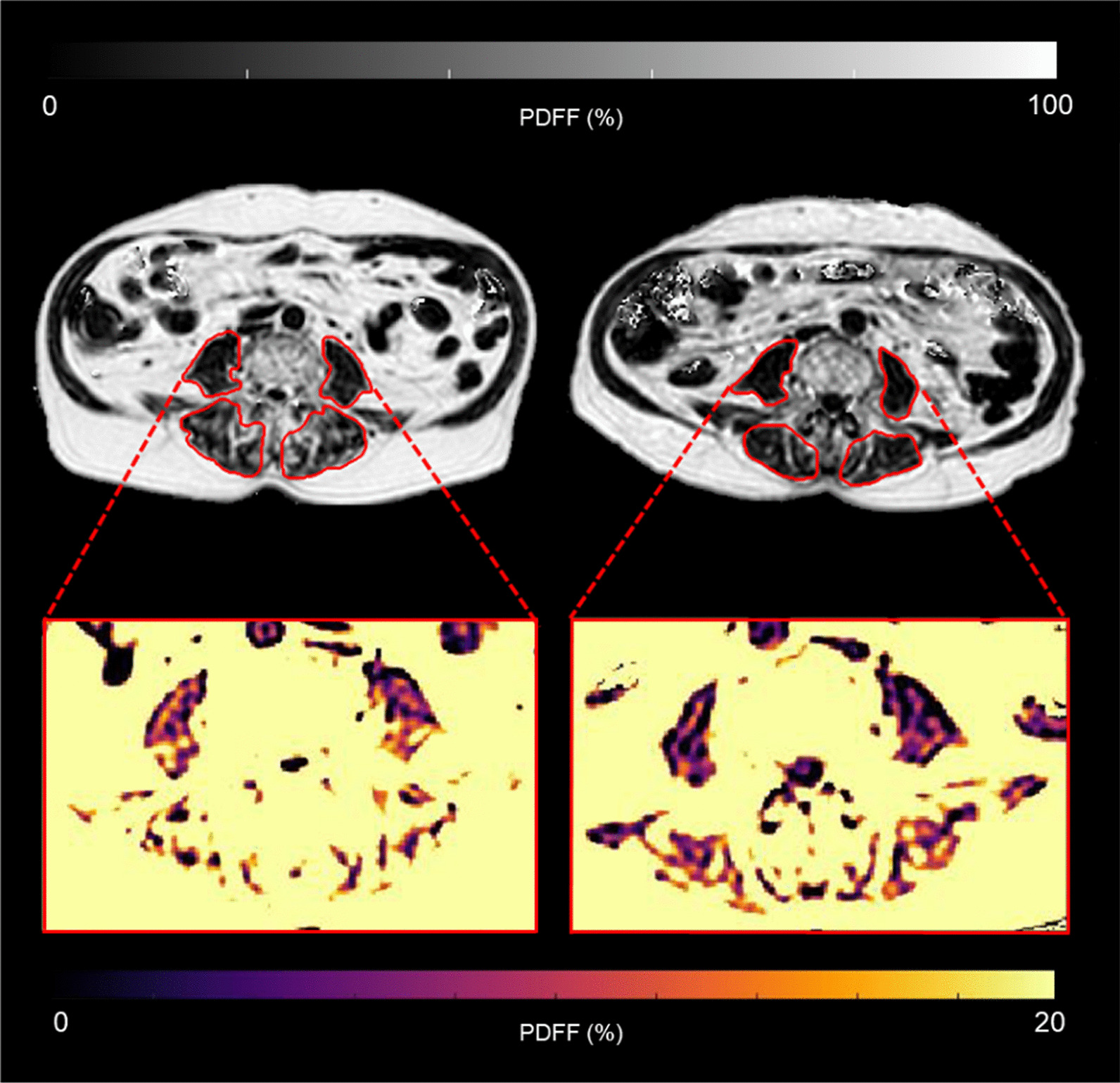
Skeletal paraspinal muscle changes in a patient with cancer cachexia: axial abdominal MRI scan of a 74 year-old patient with squamous cell carcinoma of the esophagus at baseline (left image) and follow-up after 335 days (right image). Relative muscle volume change was − 19.9% for the erector spinae and − 26.3% for the psoas muscle. Relative change of contractile tissue volume was − 19.4% in erector spinae muscle and 25.3% in psoas muscle. Relative change in fat volume was − 21.2% in erector spinae muscle and 27.7% in psoas muscle. Absolute PDFF (%) difference was 0.5 in erector spinae muscle and 1.3 in the psoas muscle. BMI decreased from 24.1 to 21.1 kg/m^2^. *BMI* body mass index,* PDFF* proton density fat fraction

A loss of skeletal muscle volume and decrease in muscle quality has been observed with MRI in cachectic cancer patients [89–91] (Fig. 3). The thigh muscle cross-sectional area was found to be reduced in patients with malignant glioma after surgery [92]. It was demonstrated that loss of muscle volume did not lead to a loss of muscle functionality in patients with gastrointestinal cancer [89]. In cachectic men suffering from gastrointestinal cancer, a greater decline in lower limb muscle mass, quality and function was found than in women with the same condition [91].

MRI-derived muscle signal intensity as a semi-quantitative measure of myosteatosis in periampullary cancer patients was found to correlate with muscle attenuation assessed with CT [93]. In women with upper gastrointestinal malignancies, increased fat infiltration of the quadriceps muscle was observed [90]. The same study also reported a less homogenous muscle composition in tumor-bearing women than in a healthy control group.

Single-slice analysis of the fat-free muscle area in patients undergoing radioembolization of colorectal cancer liver metastasis revealed a prognostic value on overall survival [94]. Thereby, low fat-free muscle area was associated with shortened overall survival. The same was reported for patients with hepatocellular carcinoma who underwent radioembolization [95]. Temporal muscle thickness, that can be readily assessed on clinical routine MR images, was found to be a predictor of survival in non-small cell lung cancer or breast cancer patients with recently identified brain metastasis [96]. In breast cancer patients, a correlation between psoas muscle area on CT scans and pectoralis major muscle area on MRI was observed [97]. These findings underline the usefulness of the pectoralis muscle area as a surrogate marker to estimate whole-body muscle mass and patient outcome and survival.

## Imaging the white adipose tissue

**Fig. 4:**
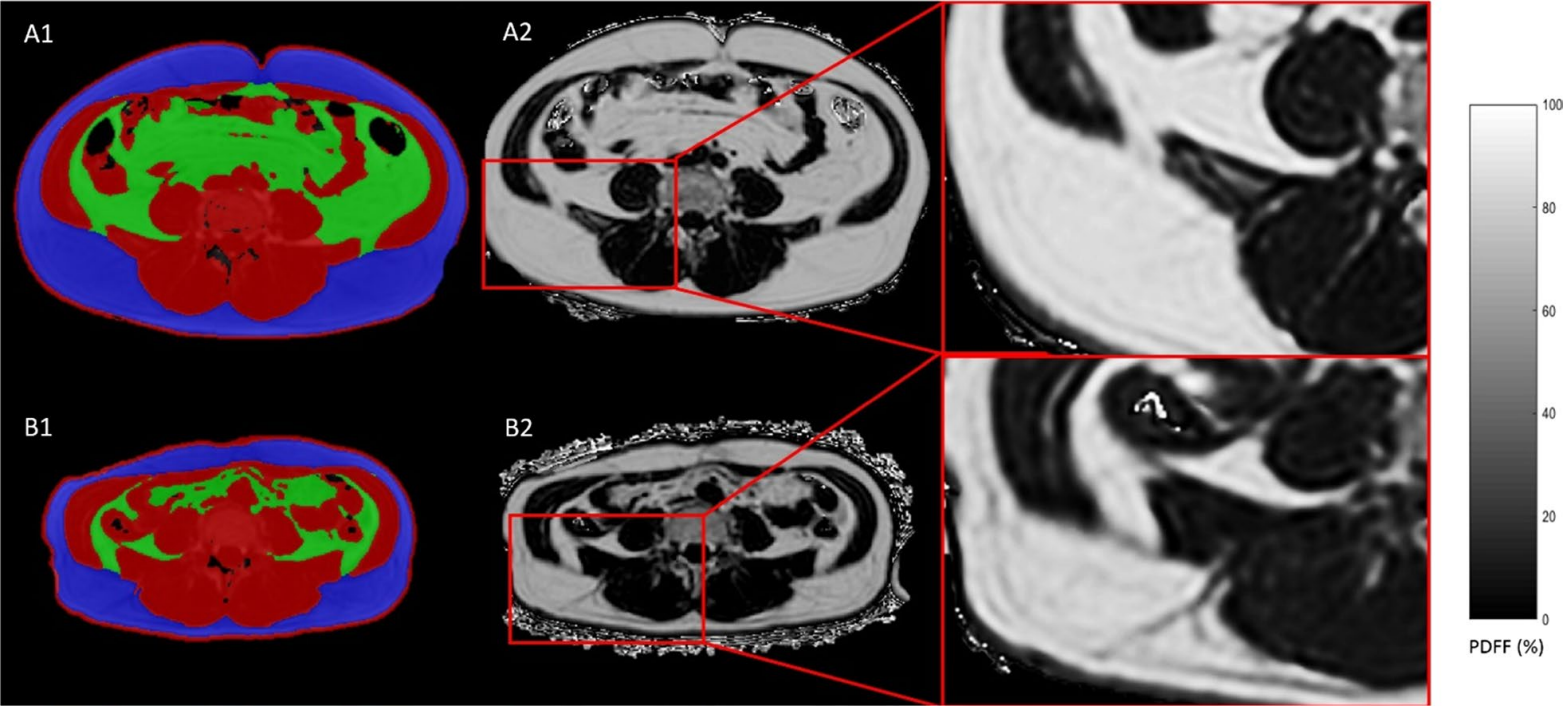
Adipose tissue changes in a patients with cancer cachexia: axial abdominal MRI images of a 54 year-old male subject with esophageal cancer at baseline (**A**) and follow-up after 8 months (**B**). **A1**, **B1** Color-coded are VAT (green), SAT (blue) and non-adipose tissue (red). **A2**, **B2** Corresponding PDFF maps showed a decrease in VAT from 83 to 72% and SAT from 87 to 76%. The BMI decreased from 30.2 to 24.1 kg/m2. *BMI* body mass index,* PDFF* proton density fat fraction,* VAT* visceral adipose tissue,* SAT* subcutaneous adipose tissue

To date, research on imaging WAT in cancer-associated cachexia remains limited. The loss of WAT, which can be further subclassified into visceral- (VAT) or subcutaneous adipose tissue (SAT), is considered to predate skeletal muscle loss in cancer cachexia. When compared to weight-stable cancer patients, individuals with cancer cachexia exhibit a reduced adipose tissue mass [98] (Fig. 4). WAT loss also predicts poorer prognosis in advanced cancer patients and is accelerated in the final months before death [99, 100]. It is therefore pertinent to monitor WAT alterations in cancer patients to detect cachexia earlier and facilitate intervention and management in palliative care. This is of particular relevance as cachexia affects most terminal cancer patients: particularly patients with lung, pancreatic, liver and gastrointestinal carcinoma where some studies reported a cachexia incidence of up to 50%. [101, 102]

Whole-body and regional distribution of WAT has been assessed with DXA in cancer patients. A longitudinal observational study on palliative cancer patients predominantly with gastrointestinal tumors reported increasing WAT loss in all body compartments with disease progression, even with maintenance or increase of caloric intake [23]. These body fat losses preceded preferentially in the trunk before other appendicular regions. Interestingly, while WAT reduced in these patients, lean tissue was maintained or even increased over the follow-up period. Furthermore, WAT changes were more pronounced and predictive of survival than lean tissue mass. Other work on patients with locally advanced or metastatic non-small cell lung or colorectal cancer found regional DXA-analysis of fat mass at the 3rd lumbar vertebra (L3) standardized to a 1-cm-thick slice equivalent to strongly correlate with total fat mass. A close correlation of fat mass at L3 in DXA to CT measurements was observed. These findings support the application of single-imaging as a reliable and accurate representator of whole-body fat [60].

CT-based assessment of WAT changes has also shed light on cancer cachexia. In a retrospective review of patients with head and neck squamous cell carcinoma (HNSCC), CT attenuation yielded substantially higher HU values for VAT than SAT. This was most prevalent in patients with T3 or T4 [103] classified tumors. Higher CT attenuation may reflect an enhanced inflammatory and fibrotic response of the adipose tissue. The combination of high VAT HU and low VAT volume lead to worse clinical outcomes and survival [104]. Similarly, high SAT and VAT attenuation was strongly predictive of poor survival in patients with adenocarcinoma and squamous cell carcinoma of the esophagus [105, 106]. Area-based quantification of VAT and SAT at the lumbar region L2-L5 is a common procedure in CT imaging and is used to estimate VAT-SAT-ratio. In pancreatic and lung cancer, high visceral-to-subcutaneous fat ratio was a prognostic factor of poor overall survival [79, 107].

While tumors often exhibit an elevated glucose metabolism, marked variations are detectable in WAT. ^18^FDG-PET/CT has been used to calculate volume and evaluate the metabolic activity within a single fat compartment by measuring FDG uptake. SUV values can be calculated for both WAT types in PET images through referencing corresponding region of interests in CT images, typically at the level of the lumbar spine [108]. Simultaneously, CT attenuation is measured. Attenuation is positively correlated with SUVs for both SAT and VAT [106]. It is known that VAT is more active than SAT and exhibits greater FDG uptake in humans. This is thought to be caused by an increased lipogenesis in glucose metabolism and inflammatory reaction in the adipocytes [104]. Greater FDG uptake in VAT has been related to worse outcomes in patients with HNSCC or pancreatic adenocarcinoma [104, 106]. In a study of pancreatic cancer patients, those with advanced primary tumor status (T-stage) malignancy had higher CT attenuations and SUVs of VAT and SAT with worse survival. Another study on advanced-stage pancreatic carcinoma patients found conversely that SAT FDG uptake was diminished and relatively decreased when compared to visceral adipose tissue, pertaining to a reduction in lipogenesis due to decreased fat cell size. This was accompanied by a strong negative correlation to primary tumor metabolism [109]. VAT volume and FDG uptake of SAT and VAT in non-small cell lung cancer patients presented no strengthened association in predicting prognosis. However, a significant negative correlation between SAT volume and FDG uptake of tumor was found and high SAT volume was associated with better progression-free survival [110]. Thus, further studies are necessary to elucidate the incongruencies in FDG uptake of WAT to determine its impacts on oncologic patients.

To the best of our knowledge, there is no study on the use of MR or MR-spectroscopy (MRS) imaging of WAT in patients with cancer-associated cachexia. Non-human trials, however, have been reported. MRI methods measured total body adipose tissue and lean mass in transgenic model mice with hepatocellular carcinoma (HCC)-associated cachexia [111]. In vivo microcomputed tomography (μCT) imaging in these murine models quantified the hypo-intense fat compartments [111]. Analogous to the HCC mouse models, CT analysis in human HCC patients showed a reduction in visceral adipose tissue [111]. In vivo MRI techniques allowed the monitoring of WAT mass changes in tumor-induced weight loss. Mice were injected with Lewis lung carcinoma or B16 melanoma cells and a control group was compared to genetically modified groups with adipose triglyceride lipase (ATGL) or hormone-sensitive lipase (HSL) deficiency. By quantifying the WAT using MRI, they confirmed the amelioration of certain aspects of cancer cachexia through lipase deficiency and its protective effect on the loss of WAT [112].

## Imaging the brown adipose tissue

BAT is found primarily in the supraclavicular region in human adults. Heat is generated there in the mitochondrial membrane by glycolysis and beta-oxidation, which is triggered by sympathetic nerval innervation. This happens as a response to cold exposure or post-prandially [113, 114]. In the context of cancer cachexia, where energy expenditure surmounts energy intake, it was believed that thermogenesis contributed to energy wasting. Increased energy expenditure via brown fat thermogenesis was observed even in absence of cold in cancer mice models [24]. Both Parathyroid hormone-related protein (PTHrP) [25] and IL6 [115] has been reported to induce browning of white fat in certain cancer types and neutralization of both hormones preserved fat mass [17, 115, 116]. Browning of WAT in adipose tissues depots outside the typical BAT storage depots have been reported in cancer-cachexia mouse models, as well as in cancer patients, by immunohistochemical staining [24]. This tissue is then oftentimes referred to as beige or brite adipose tissue. The increase in UCP1-expression in WAT, of regions such as intestinal fat, was explained by the increase of inflammation markers and IL6 [24].

^18^FDG-PET detects regions in the body with active glycolysis and is widely used for cancer diagnosis and for staging of tumors. In this context, active brown fat was perceived as nuisance signal and prevention of its activation during ^18^FDG-PET imaging was strived for [117]. Only after the discovery of brown fat in a significant number of human adults, targeted imaging was conducted to examine BAT mostly in healthy subjects. To confirm the hypothesis of hypermetabolic brown fat in cancer patients, several retrospective studies using combined ^18^FDG-PET/CT data in certain cancer types had been conducted. Some suggest a positive correlation between cancer and BAT activity [118–121], while others could not confirm the relationship [122]. A more recently published retrospective study included a larger patient number and various cancer types but could not replicate the results either [123]. BAT activation level was graded by radiologists. However, the causal inference analysis yielded only a link between outside temperature and BAT activation status, but no relation of BAT activation with cancer progression. A limitation of the aforementioned study is that BAT activation was not induced, and the limitation of retrospective studies in the context of BAT is that BAT activation was not controlled and very different baseline conditions such as season and outside temperature would bias comparability of data.

^18^FDG-PET/CT can only visualize active glycolysis in BAT and is therefore unable to detect currently non-active BAT or thermogenesis via beta oxidation. In other words, if glycolysis is not triggered in BAT during the examination, ^18^FDG-PET is little sensitive in detecting BAT. Various MRI contrast mechanisms have been used to detect brown fat presence and activation in rodents and humans [124]. However, due to many remaining challenges of traditional water-fat imaging and the limited accessibility of pioneering work such as Xenon-based MRI, these studies are still limited to mice and healthy human cohorts [124]. One work relating cachexia in murine pancreatic ductal adenocarcinoma has suggested using T_2_* contrast and fat fraction images for evaluating BAT activation status and the change in brown fat volume, respectively [125].

In summary, in vivo BAT quantification studies using ^18^FDG-PET are challenging and have found contradictive results in cancer cachexia because retrospective studies could not account for confounding factors for BAT activation and because ^18^FDG-PET only detects glycolytic activity of BAT. Future studies should control confounding factors [122, 126] and use standardized scan and BAT activation protocols. The statistical analysis should be adjusted for age, sex, BMI, physical exercise habits, hormonal status and caffeine consumption, among others. Environmental factors that influence BAT activity include the season and the current outdoor temperature. Therefore, images should be acquired during similar baseline conditions. Finally, the investigated patient cohort should consist of homogeneous cancer patient cohorts as disease progression and cachexia development highly depends on the type of cancer.

## Imaging other organs

While available studies primarily focused on the classical cachexia-related tissue compartments, we briefly review here the use of imaging for studying other organs beyond the primary tumor, the skeletal muscle, and the adipose tissue.

Recent work has uncovered a link between liver uptake of ^18^FDG and survival in cancer patients (Nakamoto et al.) [127], highlighting the potential of PET analysis as a tool for nuclear medicine physicians to infer cachexia risk. An extremely limited ^18^FDG uptake (SUVmean ≤ 1.78) of the liver substantially increases the risk of developing cachexia, leading to worse overall survival [127]. An even greater risk poses the presence of viable and/or recurrent malignant lesions on ^18^FDG-PET. These patients commonly presented with anemia, impaired liver function, systemic inflammation and poor nutritional status, characteristics which also predominate the cancer cachexia population.

Changes in visceral organ size have been monitored using quantitative CT image analysis in patients with advanced colorectal cancer. An exponential increase in liver volume, hepatic metastases, and an increase in spleen volume concurrent to muscle and fat loss were recorded in patients until death [128]. The percentage of estimated fat-free mass occupied by the liver increased in this group from 4.5 to 7.0%. The extensive organomegaly in these patients likely contributes to increased catabolism and energy expenditure (a cumulative increase of 17,700 kcal in the liver in the final 3 months of the simulation) when compared to healthy references, leading to rapid weight loss and suggests why an increased dietary energy intake as a compensatory treatment method often proves ineffective.

Analyses of ^1^H MRS of brains of cachectic pancreatic tumor-bearing mice have identified a unique "cachexia brain metabolic signature" characterized by changes to the levels of metabolites, specifically an increase in cholines and a decrease in glutamine and formate [129]. These alterations may disrupt existing neurotransmitter pathways, compromising normal brain function and increasing morbidity in cachexia-inducing pancreatic cancers. Therefore, brain spectroscopy may propose a useful noninvasive marker for predicting cachexia and should be further investigated in human patients.

## Comparison of imaging modalities in cancer cachexia

CT and MRI are accepted as gold standard methods to determine body composition (Table 1). They enable the discrimination between changes in fat depots, specific muscles, and organs [60, 100]. MRI is safer for one decisive reason: there is no ionizing radiation exposition. Since repeated measurements are essential for tracking cancer progression, no further health risks are associated with frequent image acquisition using MRI, making it a highly plausible alternative for monitoring cachectic cancer patients [130]. With water-fat MRI, lean soft tissue, adipose tissue and diffuse fatty infiltration of organs can be precisely defined. The measurement error for MRI-based cross-sectional area of appendicular skeletal muscle and -SAT is reported to be at 2%, making it a reliable approach for estimating appendicular tissue distribution in vivo [130]. However, MRI is not as widely accessible as CT. It is time-demanding and is subject to greater financial costs [131]. Cross-sectional CT analysis of skeletal muscle area at lumbar landmarks, specifically L3, are strongly related with whole-body fat and fat-free compartments in cancer patients [60]. As such, regional CT-based evaluation is applicable for estimating whole-body changes in patients [60].

**Table 1:** Summary of the uses of imaging modalities in diagnosis, monitoring and prognosis and findings in cancer cachexia for different cancer types

Imaging modality	Findings	Cancer type	References
Dual-energy X-ray absorptiometry (DXA)	Muscle mass and body fat loss observed in advanced cancer patients Progression of muscle mass loss was greater in men compared to women		[51, 52]
	67% of palliative cancer patients had a low appendicular lean soft tissue index		[53]
	Accelerated depletion of body fat was found compared to lean tissue, with lean tissue loss in the arms but a relative weight gain in the trunk		[54]
	WAT loss increased with disease progression, preferentially in the trunk before appendicular regions, despite the maintenance or increase of caloric intake	Gastrointestinal cancer	[23]
Computed Tomography (CT)	Sarcopenia associated with higher mortality and morbidity rates in cancer patients		[63–66]
	High muscle radiodensity was a prognostic factor for longer survival	Non-small cell lung cancer	[69]
	Low muscle attenuation of cross-sectional paraspinal muscles predictive of unsatisfactory therapy response	Renal cell cancer	[73]
	Low skeletal muscle indices strongly associated with prevalence of dose limiting chemotherapy related toxicity		[74–78]
	Skeletal muscle loss during neoadjuvant chemotherapy predictive of increased postoperative mortality	Esophageal cancer	[81]
	Myosteatosis related to shorter survival and systemic inflammation Higher myosteatosis levels related to longer hospitalization times	Colorectal cancer, Pancreatic cancer, Distal cholangiocarcinoma	[85–87]
	Higher VAT CT attenuation than SAT may indicate inflammation and fibrotic response High VAT HU and low VAT volume lead to worse clinical outcomes and survival	Head and neck squamous cell carcinoma	[104]
	Higher VAT and SAT CT attenuation lead to poor survival	Esophageal adenocarcinoma and squamous cell carcinoma	[105, 106]
	High VAT/SAT ratio prognostic of poor overall survival	Pancreatic cancer, Lung cancer	[79, 107]
	Decrease in fat mass and fat-free mass post neoadjuvant chemotherapy but relative increase in sarcopenic obesity prevalence	Respiratory and gastrointestinal tract cancer	[65, 137]
	Exponential increase in liver volume, hepatic metastases and increase in spleen volume was observed concurrent to muscle and fat loss	Advanced colorectal cancer	[128]
Magnetic resonance Imaging (MRI)	T_2_* contrast and fat fraction imaging a possible method to evaluate BAT activation status and brown fat volume change	Murine pancreatic ductal adenocarcinoma	[125]
	Loss of skeletal muscle volume und muscle quality observed in cachectic cancer patients		[89–91]
	Reduction in cross-sectional area after surgery	Malignant glioma	[92]
	Greater decline in lower limb muscle mass, quality and function in men than women	Gastrointestinal cancer	[91]
	Increased fatty infiltration of quadriceps muscle Lower homogeneity in muscle composition	Upper gastrointestinal cancer	[90]
	Low fat-free muscle area associated with shorter overall survival	Colorectal cancer, Hepatocellular carcinoma	[94, 95]
	Temporal muscle thickness predictor of survival in patients with brain metastasis	Non-small cell lung cancer, Breast cancer	[96]
	Amelioration of aspects of cancer cachexia through lipase deficiency with protective effects on WAT loss	Lewis lung carcinoma, B16 melanoma	[112]
	Increase in cholines and decrease in glutamine and formate in ^1^H MRS analyses of brains of cachectic mice	Pancreatic cancer	[129]
^18^FDG-PET	Increased ^18^FDG uptake in cachexia-inducing tumors compared to non-cachectic tumors in mice		[39]
	Metabolic tumor volume positively correlated with the degree of weight loss	Gastric cancer	[41]
	PET/CT based radiomics analysis of primary tumor and skeletal muscle could predict probability of cachexia onset before therapy	Advanced non-small-cell lung carcinoma	[42]
	Elevated tumor activity associated with greater risk of malnutrition however no correlation with CT-measured body composition	Lung cancer	[43]
	Tumor mass and percentage of anaerobic metabolism contribute to greater energy burden, with consequent increase in muscle wasting and negative energy balance		[44]
	Elevated ^18^FDG uptake in VAT related to worse outcomes	Head and neck squamous cell carcinoma, Pancreatic adenocarcinoma	[104, 106]
	High SUV of VAT and SAT lead to worse survival SAT ^18^FDG uptake was reduced and relatively decreased compared to VAT and correlated negatively correlated with primary tumor metabolism	Pancreatic cancer	[109]
	SAT volume negatively correlated with ^18^FDG uptake of tumor High SAT volume associated with better progression-free survival	Non-small cell lung cancer	[110]
	No relation of BAT activation with cancer progression		[123]
	Reduced liver ^18^FDG uptake increased the risk of cachexia and worse overall survival		[127]

*VAT* Visceral adipose tissue, *HU* Hounsfield units, *SAT* Subcutaneous adipose tissue, *BAT* Brown adipose tissue, *WAT* White adipose tissue, *SUV* Standardized uptake value

Many clinicians are limited to using only single-slice 2D analysis due to the time-consuming nature of manual segmentation and correction of tissue boundaries. However, single 2D slices are prone to greater variability in volume calculation than a series of images. Recent advancements in artificial intelligence (AI) have allowed for the conversion of CT and MRI images into mineable data that can be accessed for quantitative features analyses. This process called radiomics offers an automated three-dimensional (3D) approach of whole-body tissue assessment. The transition from manual 2D segmentation to automated 3D volumetric assessment using AI-based methods can thus improve the accuracy in determining body composition and more importantly allow for a better individualization of treatment plan for cancer patients [131].

DXA is overall a fast technique for assessing body composition but lacks the precision of the aforementioned imaging modalities (Table 1). It is also not readily available or implemented in cancer settings. CT imaging outweighs DXA in this regard as it is already routinely acquired during the staging and follow-up assessments of cancer patients [60]. DXA is a projection technique and cannot assess changes in individual muscle groups such as myosteatosis, described as abnormal skeletal muscle fat infiltration and considered a marker for muscle quality [132, 133]. It is however simple, highly reproducible and therefore practical for repeated control measurements. It requires lower radiation doses than CT and can differentiate easily between lean body mass, adipose tissue and bone mineral content. For determining lean body mass in follow-up measurements of patients with worsening disease progression, as is often the case in cachexia cancer, whole-body DXA may not be reasonable. This is because it does not take into account for small changes in fluid distribution in the tissues [23]. Changes in the fluid status such as dehydration or edema cannot be differentiated from lean soft tissue with DXA and therefore hinder exact estimation of lean soft tissue mass and composition [134, 135]. Also, tumor mass and lean tissue mass cannot be discriminated with certainty, and a decreased precision in obese patients is known [136]. A further limitation of DXA is the inability to segregate VAT from SAT. When evaluating cachectic cancer patients, VAT imaging delivers useful prognostic information that should be considered in addition to clinical changes in physical appearance or fatigue and weight loss. To this accord, CT and MRI are able to determine the adipose tissue changes and body composition in vivo with extreme precision [60].

Nevertheless, DXA is relevant for cancer intervention because the relative proportions of LBM and adipose tissue impact the degree of chemotherapy-induced toxicity. Previous work has demonstrated the impacts of chemotherapy on body composition: although generally fat mass and fat-free mass were shown to decrease after neoadjuvant chemotherapy, a relative increment in patients with sarcopenic obesity was observed, and sarcopenic obesity was an important independent predictor of survival in patients with respiratory and gastrointestinal tract tumors [65, 137]. Given the low sensitivity of BMI in detecting finer changes in body composition, clinicians are more likely to overlook these kinds of anomalies, so studying body composition could be advantageous. Low LBM is dominant in cachectic cancer patients—a depletion of LBM leads to a lower volume of distribution for hydrophilic drugs—and is linked to greater chemotoxicity [138, 139]. Low LBM percentage can even lead to a decrease in serum creatinine blood levels, an overestimation of renal filtration and consequent misdosage of chemotherapeutic medication, eliciting severe toxicity [140]. Identification of abnormal changes to body composition distinctive to cancer-cachexia patients would therefore help prevent such ramifications during treatment.

^18^FDG-PET describes the metabolic activity of adipose tissue compartments [141]. ^18^FDG-PET/CT not only provides anatomical but important functional information on body tissues. ^8^FDG -uptake of AT could be a valuable qualitative imaging biomarker for fat analysis. However, there is currently no standard method for SUV of WAT. This could explain some of the contradictive results presented by studies on cancer patients where measurements were based either on a single-slice region of interest or whole tissue assessments using multiple slices [106]. An agreement on a conform approach in the future is needed.

## Outlook

There is an urgent need of impetus to find better treatment options for cachexia patients. Progress is certainly being made in this domain, however much work remains, particularly in advancing from an ad hoc treatment approach toward identifying methods of early detection and prevention. As reviewed in this piece, an array of imaging methodologies is capable of delineating aspects of the disease, however often fall short as comprehensive standalone diagnostic tools.

Biomarkers obtainable from circulation are often of high diagnostic value, while keeping invasiveness low. Blood-borne biomarkers can be subdivided into 4 groups; Cachexia-inducing, inflammation related, skeletal muscle and adipose tissue wasting markers or cachexia-related micro-RNAs and have been reviewed in detail elsewhere [142]. These biomarkers may originate either from the primary tumor itself, or from the host in response to the tumor and metabolic alterations. Unfortunately, there is an immense degree of variability to the applicability of each biomarker, as many blood biomarkers are adequate only for a few or only a single type of cancer and vary significantly depending on muscle loss and gender [143, 144]. This poses the question as to whether or not combined biomarker screens may be advantageous. Reassuringly, a current study has shown that a panel of cachexia biomarkers did indeed accurately reflect cachexia prevalence and weight loss across 12 different cancer types [145]. It is unclear though, whether these biomarkers simply reflect a diseased state, or could also indicate disease progression and degree.

Many of the current cachexia blood-borne biomarkers are simultaneously potential therapeutic targets. Unfortunately, if a biomarker is targeted in a therapeutic manner, this diminishes its value as a marker of disease progression/state, which would be necessary to measure therapeutic success. The imaging biomarkers described in this review on the other hand, remain unperturbed by therapeutic intervention in the short term and so reveal themselves as powerful tools in the clinical setting of cancer cachexia. Future clinical studies may employ suitable imaging techniques to measure the outcome of cachexia therapies, providing valuable insight into body composition and skeletal muscle and adipose tissue architecture.

Therefore, although blood-borne biomarkers are an important piece of the cachexia puzzle, it is clear that alternative methods of detection are necessary. Combining blood-borne biomarkers with the described innovative imaging biomarkers may yield promising therapeutic strategies. To further reveal this potential, future clinical studies with a focus on imaging biomarkers are desperately needed in the context of cancer cachexia.

Noninvasive imaging enables the multi-organ assessment of cachexia effects in cancer patients. However, despite the recent successes, imaging currently only helps in part in pathophysiology understanding and has not provided a clear widely used biomarker for cachexia risk assessment. CT techniques will remain most probably the most popular approach for assessing cachexia given the wide use of CT in the staging of many oncological entities. However, as many oncological centers use more MRI exams, the use of MRI for assessing body composition and organ-specific changes is expected to increase in cancer cachexia. When MRI or CT is already performed in the clinical work-up of the patient, an imaging analysis of the cachectic phenotype could be combined with the main diagnostic reporting, especially when facilitated by the use of automated analysis tools. However, more clinical studies using modern imaging methods in answering cachexia development-driven questions based on large-scale prospective and homogeneous cohorts are needed toward establishing quantitative CT and MR imaging biomarkers for cachexia early prediction.

## Conclusion

Cancer remains a leading cause of death in all countries and its stable increase in incidence and mortality is concerning. Although imaging of body composition change in cancer is well documented, few studies have informed us on the niche of cachectic cancer patients. This owes partially to the wide variations in clinical presentation and the absence of a validated international consensus for the diagnostic criteria of cancer-associated cachexia. While cancer cachexia is principally characterized by involuntary weight loss, it can also manifest with a constellation of other medical conditions such as malnutrition, anorexia or systemic inflammation [146]. It occurs at different disease states and varies with cancer type and stage [3]. Indeed, many definitions have been proposed but do not well accommodate the complexity of this syndrome in a manner for it be integrated into practice with enough certainty. Depending on the definition used, the prevalence of cachexia in a population of cancer patients can vary from 12 to 85% [53] which jeopardizes the comparability of studies. This inconsistency impedes oncologists to systematically diagnose and monitor cancer-cachexia.

Overwhelming evidence suffices the competencies of imaging tools to measure body composition and delineate tissue compartments. CT and MRI stand here as favorable approaches that produce high spatial and contrast resolution images [130]. We need imaging to investigate different body types and accumulate a comprehensive understanding of the processes involved in patients with cancer-cachexia, with attention to factors such as cancer type, stage, and current therapy. This comes with the caveat that further studies should be conducted based on a holistic definition and classification system of cancer cachexia. Only then can we solidify how cachexia imaging can be best implemented into clinical routine.

## Data Availability

Not applicable.

## Abbreviations

AI: Artificial intelligence
APR: Acute-phase response
ATGL: Adipose triglyceride lipase
BAT: Brown adipose tissue
BMI: Body mass index
CT: Computed tomography
^18^FDG-PET: (^18^F) fluoro-2-deoxy-D-glucose Positron emission tomography
HCC: Hepatocellular Carcinoma
HNSCC: Head and neck squamous cell carcinoma
HSL: Hormone-sensitive lipase
UCP1: Uncoupled protein 1
LBM: Lean Body Mass
L3: 3^Rd^ lumbar vertebra
MRI: Magnetic resonance imaging
MRS: Magnetic resonance spectroscopy
PET: Positron emission tomography
PDFF: Proton density fat fraction
PTHrP: Parathyroid hormone-related protein
SAT: Subcutaneous adipose tissue
SUV: Standardized uptake value
VAT: Visceral adipose tissue
WAT: White adipose tissue
μCT: Microcomputed tomography
2D: Two-dimensional
